# L-Carnitine potentiates the anti-inflammatory and antinociceptive effects of diclofenac sodium in an experimentally-induced knee osteoarthritis rat model

**DOI:** 10.22038/ijbms.2020.43136.10138

**Published:** 2020-08

**Authors:** Suzan A. Khodir, Marwa A. Al-Gholam, Heba R. Salem

**Affiliations:** 1 Medical Physiology Department , Faculty of Medicine, Menoufia University, Shebin El-Kom, Menoufia, Egypt; 2 Anatomy and Embryology Department, Faculty of Medicine, Menoufia University, Shebin El-Kom, Menoufia, Egypt

**Keywords:** CGRP, COX-2, L-carnitine, MMP-13, Osteoarthritis

## Abstract

**Objective(s):**

The aim of the present research is to investigate the efficacy of L-carnitine (LC) as a complementary therapy to diclofenac sodium (Dic) treatment in a mono-iodoacetate (MIA) induced knee osteoarthritis (OA) rat model, with respect to pain relief and the underlying pathology.

**Materials and Methods:**

Fifty adult male albino rats were randomly divided into five groups (n=10): Control, OA, OA/Dic, OA/LC, and OA/Dic+LC. Knee diameter and pain assessment tests were done weekly. After four weeks, serum malondialdehyde, reduced glutathione, interleukin 1-β, tumor necrosis factor-alpha, prostaglandin E2, and bone-specific alkaline phosphatase were measured. The injected knees were removed and processed for the histological and immunohistological study of matrix metalloproteinase-13 (MMP-13) and cyclooxygenase 2 (COX-2). Also, histological examination of dorsal root ganglia and calcitonin gene-related peptide (CGRP) expression in the spinal cord were assessed.

**Results:**

Treatment with Dic and/or LC significantly reduced knee swelling, improved pain-related behaviors, inflammatory and oxidative stress markers, attenuated the MIA-mediated histopathological alteration in the knee joint, and down-regulated expression of MMP-13 and COX-2 in the knee joint. It, also, significantly reduced CGRP expression, compared with the OA group. Dic+LC showed a better effect in improving some parameters than each treatment alone.

**Conclusion:**

LC plus Dic is a more effective therapy than Dic alone for OA treatment.

## Introduction

Knee osteoarthritis (OA), a progressive degenerative joint disease, is one of the leading causes of disability worldwide. It can result in impaired quality of life and increased health costs (1). Pain is the major symptom of OA; however, the current analgesics including nonsteroidal anti-inflammatory drugs are unsatisfactory in some patients, and their long-term use produces several side effects. Diclofenac sodium (Dic) is a widely used nonsteroidal anti-inflammatory drug for OA treatment. Diclofenac exerts its action via inhibition of prostaglandin synthesis by inhibiting cyclooxygenase-1 (COX-1) and cyclooxygenase-2 (COX-2) with relative equipotency. However, it fails to adequately improve the pathophysiological and biochemical mechanisms involved in cartilage degeneration (2). Therefore, the search for other effective treatments with more safety is needed. 

L-Carnitine, the biologically active form of carnitine, is synthesized from the essential amino acids lysine and methionine (3). L-carnitine is a powerful antioxidant molecule with an anti-inflammatory effect as shown in different experimental models (4, 5). Thus, it had attracted attention for controlling OA in experimental studies (6). 

The pathologic mechanisms underlying OA-induced pain are not well understood, but it has been established that peripheral and central mechanisms are involved (7, 8). The inflammatory mediators, involved in pain pathogenesis include prostaglandin E2 (PGE2), interleukin-1-beta (IL-1β), and tumor necrosis factor-alpha (TNF-α). These mediators increase the secretion of matrix metalloproteinases (MMPs; matrix-destructive enzymes) (9). The central mechanisms involve the affection of peripheral afferent and dorsal root ganglion (DRG) neurons, and the sensitization of pain perception (10). Spinal neuropeptides, such as calcitonin gene-related peptide (CGRP), can modulate OA-induced pain. High CGRP level has been reported to produce hyperalgesia (11). 

Knee OA can be experimentally induced in rodents by intra-articular injection of a mono-iodoacetate (MIA), a chondrocyte glycolytic inhibitor (12). This model produces pathophysiological changes and significant pain-related behavior similar to that of human OA (13).

The aim of this study is to evaluate the efficacy of LC as a complementary therapy to Dic in the MIA-induced knee OA rat model, with respect to pain relief and the underlying pathology. Also, the impact of MIA-induced OA on CGRP expression in the spinal cord has been investigated to decide whether it has been improved by LC and/or Dic treatment or not as a suggested mechanism of action. To the best of our knowledge, this is the first study to investigate the complementary effect of LC to the standard Dic in the MIA-induced OA rat model. 

## Materials and Methods

***Animals***

Fifty adult male Sprague Dawley albino rats, weighing 180±20 grams each, were used in this study. Rats were housed in standard conditions with a natural light-dark cycle and were fed a standard rat chow with free access to water. Rats were left to acclimatize for one week before the experiment. The experiment was approved by the Research Ethics Committee, Faculty of Medicine, Menoufia University, Egypt. 

***Experimental design***

Rats were randomly divided into five groups (10 rats each):

1. Control (C) group: Rats were injected with 50 µl normal saline once intra-articularly through the infrapatellar ligament of the left knee joint. 

2. Osteoarthritis (OA) group: OA was induced (pre-anesthetized with ether) by single intra-articular injection of MIA (3 mg/50 µl, diluted in normal saline, Sigma, St Louis, MO, USA) using a 26-gauge syringe through the infrapatellar ligament of the left knee joint (14). Animals with warmth, swelling, and tenderness as compared with the opposite knee joint were judged to have OA (14).

3. Osteoarthritis/Diclofenac sodium treated (OA/Dic) group: Osteoarthritic rats received diclofenac sodium (5 mg/kg/day, dissolved in saline, Voltaren^®^; Novartis, Egypt) orally for 4 weeks (15).

4. Osteoarthritis/L-carnitine treated (OA/LC) group: Osteoarthritic rats received LC (100 mg/kg/day, L-carnitine syrup; Mepaco, Egypt) orally for 4 weeks (6).

5. Osteoarthritis/Diclofenac sodium+L-carnitine treated (OA/Dic+LC) group: Osteoarthritic rats received diclofenac sodium (5 mg/kg/day) (15), and LC (100 mg/kg/day) orally for 4 weeks (6). 

All treatments started one day after OA induction. Pain assessment tests were done weekly. At the end of the experiment, blood samples were collected for subsequent biochemical analysis. Thereafter, all rats were anesthetized using ketamine (100 mg/kg and xylazine, 10 mg/kg, IP) and decapitated. Lumbar spinal cord with ipsilateral dorsal root ganglia (DRG) (levels L2-L5) were quickly dissected out. Samples for real-time PCR analysis of CGRP were stored at -80 ˚C and DRG samples were prepared for histological and immunohistochemical analyses. Also, the left knees of the rats were removed and processed for histological and immunohistological assessments. 

***Knee diameter***

Knee diameters were measured to assess joint swelling as an indicator of inflammation. The diameters of both knees were measured with a manual caliper on days 0 (before MIA injection), 1, 7, 14, 21, and 28 (post-injection). Results were presented as the difference between ipsilateral and contralateral knee diameters (16).

***Pain assessment methods***

*Knee bend test*

The knee bend test was used to assess the movement-induced nociception. Briefly, the squeaks and/or struggle reactions in response to five alternative flexions and extensions of the knee joint (performed within the physiological limits of knee movements) were recorded. The scoring was determined as follows: score 0: no responses, score 0.5: struggle to maximal flexion/extension, score 1: struggle to moderate flexion/extension or vocalizations to maximal flexion/extension and score 2: vocalizations to moderate flexion/extension. The sum of the animal’s reactions, with maximal values of 20, represents the knee bend score (17).

*Rotarod performance test*

Using an accelerating rotarod performance test, the time to fall was measured. The rats were exposed to an acceleration speed of 5 to 16 rpm, over one minute, before being maintained at this speed, while the time of first failure to stay atop the rod was monitored with a cutoff time of 3 min. Data are expressed as the time to fall in seconds (18).

*Treadmill exercise endurance test*

All rats were familiarized to exercise by running for 10 min/day for 4 days at a speed of 10 m/min (19). Twenty-four hours later, rats were forced to run on the motor-driven treadmill at a speed of 15 m/min until they were completely exhausted. Exhaustion was defined as the inability of a rat to run despite being placed at the front of the treadmill three times. The average time until exhaustion for each rat was estimated (20). 

*Gait scoring*

Gait analysis was performed by applying ink to the ventral surface of the rear feet of rats and allowing them to walk along the full length of a sheet of paper. Footprints made by the injected leg were compared with the noninjected one to assess weight-bearing during movement. The gait was analyzed according to the score shown in Table 1 (21). 

***Blood sampling and biochemical analysis***

At the end of the experiment, animals were fasted overnight, and then retro-orbital blood samples were collected, allowed to coagulate for 30 min at room temperature, and then centrifuged at 2000 rpm for 15 min. The serum was collected and frozen at -80 ^°^C until analyzed.

Serum interleukin 1-β (IL-1β), tumor necrosis factor-alpha (TNF-α), prostaglandin E2 (PG-E2), and bone-specific alkaline phosphatase (BALP) levels were measured using the corresponding rat enzyme-linked immunosorbent assay (ELISA) kits. (IL-1β: ab100768, Abcam, Cambridge, UK), (TNF-α: R&D Systems Inc., Minneapolis, USA.), (PG-E2: ab133021, Abcam, Cambridge, UK), and (BALP: MBS703336, MyBioSource, San Diego, USA) according to the manufacturer’s instructions. Serum malondialdehyde (MDA) and reduced glutathione (GSH) were determined using colorimetric kits (Biodiagnostic Company, Dokki, Giza, Egypt). 

***Histological and immunohistochemical analyses***

Knee joints and DRG were fixed in 10% formalin solution for 24 hr and embedded in paraffin wax. Knee joints were decalcified in 20% EDTA solution for 21 days before embedding in paraffin. For histological examination, 5 μm sections were deparaffinized and rehydrated using graded ethanol (100%, 90%, and 70%) series and stained with hematoxylin and eosin (H&E).

For scoring histological injuries of the articular cartilage, a modified Mankin’s histological score (original score proposed by Mankin *et al*., 1971) was used (22). It was performed as described in Table 2.

For immunohistochemical study, paraffin sections (5 μm) were deparaffinized, rehydrated in descending grades of ethanol, and after antigen retrieval with 10 m mol/l citrate acid solution (pH 6), specimens were preincubated with blocking solution (goat serum) for 5 min and were then incubated overnight at 4 ^°^C with the primary antibody (MMP-13, Gene Tex; COX-2, Thermo Scientific, working dilution 1:500). Sections were incubated with secondary biotinylated antibody (goat anti-mouse IgG; Sigma Aldrich, St. Louis, USA) for 20 min. The streptavidin-peroxidase complex was then applied to sections for 10 min. The secondary antibody binding was visualized by incubating sections with 3, 3 diaminobenzidinetetrahydrochloride (DAB; Sigma Aldrich, St. Louis, USA). Finally, slices were rinsed with phosphate-buffered saline, counterstained with hematoxylin and mounted.

For quantitative assessment, five different H&E stained and immunostained sections (200 x magnification) were obtained from at least five different rats from each group. The number of neurons in DRG and immunopositive cells in the fields taken from at least three sections per animal was counted using Image J software (1.74v; National Institute of Health, Bethesda, Maryland, USA), and averaged per field for each animal. 

***Real-time (RT)-PCR analysis***

Lumbar spinal cord levels L2-L5 were harvested, and relative mRNA levels of CGRP were analyzed by RT-PCR. Total RNA was isolated using the RNA easy Mini kit (Qiagen, Valencia, CA) according to the manufacturer’s instructions. Reverse transcription was performed with a programmable thermocycler (Biometra, Göttingen, Germany) using the Quanti-Tec^®^ reverse transcription kit (Qiagen) for first-strand cDNA synthesis. Then, cDNAs were amplified by PCR assays with SYBR Green Mix kits (Qiagen), and the data were analyzed using 7500 real-time PCR System (Applied Biosystems, Inc. Foster City, CA, USA). A cycle threshold (Ct) value was obtained from each amplification curve, and the relative quantification of CGRP expression was determined using the ΔΔCt method (23). The primers employed were as follows: forward primer sequence: 5’-*TCTAGTGTCACTGCCCAGAAGAGA-*3’and reverse primer sequence: 5’*-GGCACAAAGTTG TCCTTCACCACA*-3’

***Statistical analysis***

The SPSS version 16 (SPSS, Inc., USA) was used for the analysis of data. The results were expressed as mean±standard deviation (SD). The significance of differences between groups was determined by one-way analysis of variance (ANOVA) followed by *post hoc* Tukey’s test. *P-values*<0.05 were considered statistically significant. 

## Results

***Biochemical parameters***

Regarding oxidative stress markers, the OA group showed significantly higher serum MDA and significantly lower GSH levels than the C group (*P*<0.001). The treatment with Dic and/or LC caused a significant decrease in serum MDA, compared with the OA group (*P*<0.05, *P*<0.05, and *P*<0.001, respectively). Also, treatment with LC or Dic+LC caused a significant increase in serum GSH, compared with the OA group (*P*<0.001); while, the treatment with Dic alone did not show a significant effect (*P*>0.05). Dic+LC showed a better effect in reducing serum MDA and increasing reduced GSH levels than when Dic was administered alone (*P*<0.05) (Table 3). 

Regarding inflammatory markers, the OA group showed significantly higher serum TNF-α and IL1-β levels, compared with the C group (*P*<0.001). Treatment with Dic and/or LC caused a significant decrease in serum TNF-α and IL1-β, compared with the OA group (*P*<0.001). The combined treatment showed a better effect in reducing serum TNF-α than when Dic or LC were administered alone (*P*=0.001 and *P*<0.001, respectively). Also, OA/Dic+LC group showed significantly lower serum IL1-β than OA/Dic or OA/LC groups (*P*<0.05 and *P*<0.001, respectively). 

Also, there was a significant increase in serum PGE2 level in the OA group, compared with the C group (*P*<0.001). The treatment with Dic and/or LC caused a significant decrease in serum PGE2, compared with the OA group (*P*<0.05, *P*<0.05, and *P*<0.001, respectively). The combined treatment showed a better effect in reducing serum PGE2 than when Dic or LC were administered alone (*P*<0.05) (Table 3). 

Moreover, a significant increase in serum BALP level was observed in the OA group, compared with the C group (*P*<0.001). The treatment with Dic and/or LC caused a significant decrease in serum BALP, compared with the OA group (*P*<0.05, *P*<0.001, and *P*<0.001, respectively). Serum BALP level was significantly lower in the OA/LC group than in the OA/Dic group (*P*<0.05). Co-administration of LC with Dic showed a better effect in reducing serum BALP level than when Dic was administered alone (*P*<0.05) (Table 3). 

***Knee diameter***

**Figure 1:**
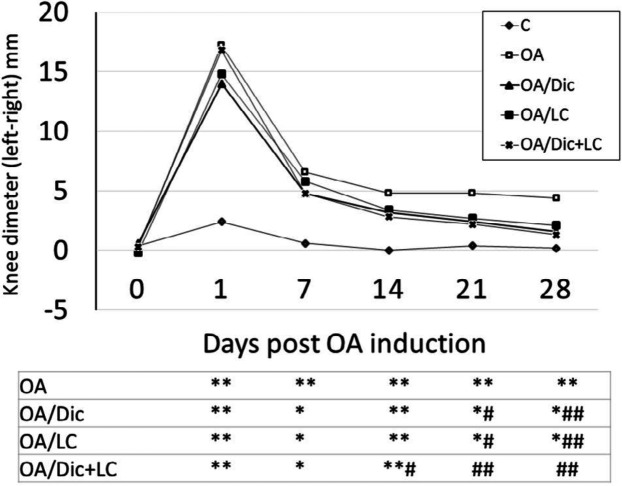
Effect of diclofenac sodium and/or L-carnitine on knee diameter (left-right) in mono-iodoacetate induced osteoarthritis rat model. * *P<*0.05, ** *P<*0.001 vs C group; # *P<*0.05, ## *P<*0.001 vs OA group

MIA injection caused a significant increase in the difference between the ipsilateral and contralateral knee diameters at all-time points, compared with the C group (*P*<0.001). The treatment with Dic or LC caused a significant decrease the knee diameter differences on days 21 and 28, compared with the OA group (*P*<0.05 and *P*<0.001, respectively); while, the combined treatment caused a significant decrease in the knee diameter difference earlier on day 14, compared with the OA group (*P*<0.05). Also, the combined treatment was able to return the values to normal (*P*>0.05 vs C group) on day 21, which was not observed with each treatment alone (Figure 1). 

***Pain assessment***

**Figure 2:**
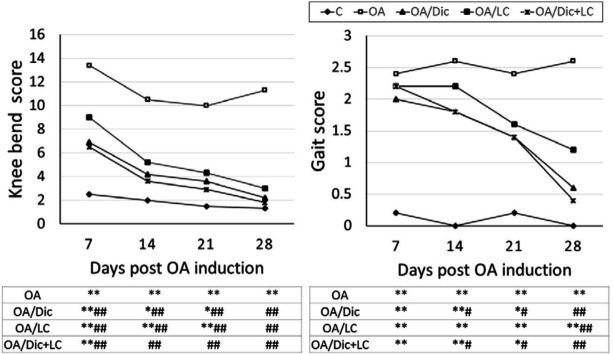
Effect of diclofenac sodium and/or L-carnitine on knee bend and gait scores in mono-iodoacetate induced osteoarthritis rat model

In the OA group, there was a significant increase in the knee-bend and gait scores at all time points, compared with the C group (*P*<0.001). The treatment with Dic and/or LC resulted in significantly lower knee-bend score values at all time points, compared with the OA group (*P*<0.001). Also, OA/Dic group showed a significant decrease in the gait score starting on day 14 (*P*<0.05); while, OA/LC showed a significant decrease on day 28 (*P*<0.001), compared with the C group. Moreover**, **the combined treatment was able to return the values of the knee-bend score to normal (*P*>0.05 vs C group) on day 14, which was not observed with each treatment alone (Figure 2). 

**Figure 3:**
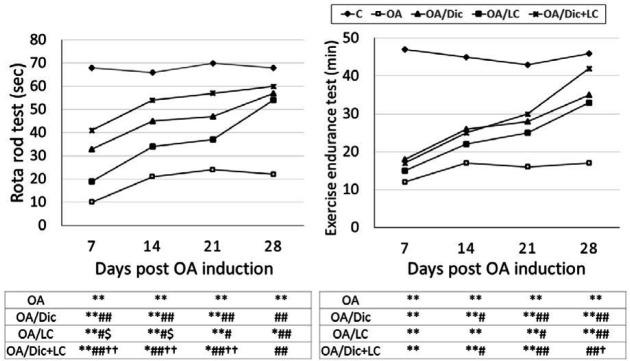
Effect of diclofenac sodium and/or L-carnitine on rotarod and exercise endurance tests in mono-iodoacetate induced osteoarthritis rat model. * *P<*0.05, ** *P<*0.001 vs C group; # *P<*0.05, ## *P<*0.001 vs OA group; $ *P<*0.05, $$ *P<*0.001 vs OA/Dic; † *P<*0.05, †† *P<*0.001 vs OA/ LC group

Rotarod analysis revealed significantly lower values in the OA group than in the C group at all time points (*P*<0.001). The treatment with Dic, LC, or combination of both showed significantly higher values at all time points, compared with OA. Treatment with Dic or Dic+LC showed a better effect than LC alone, as indicated by significantly higher values in OA/Dic on days 7 and 14 (*P*<0.05) and in OA/ Dic+LC on days 7, 14, and 21 (*P*<0.001) than in OA/LC (Figure 3). 

Exercise endurance capacity analysis revealed the significantly lower time to fatigue in the OA group than in the C group at all time points (*P*<0.001). Dic and Dic+LC treatments resulted in significantly lower values starting on day 14; while, LC treatment resulted in significantly lower values starting on day 21 compared with the OA group (*P*<0.05). OA/Dic+LC group showed significantly higher values than the OA/LC group on day 28 (*P*<0.05). The combined treatment was able to return the values to normal (*P*>0.05 vs C group) on day 28, which was not observed with each treatment alone (Figure 3).

***mRNA expression of CGRP***

**Figure 4:**
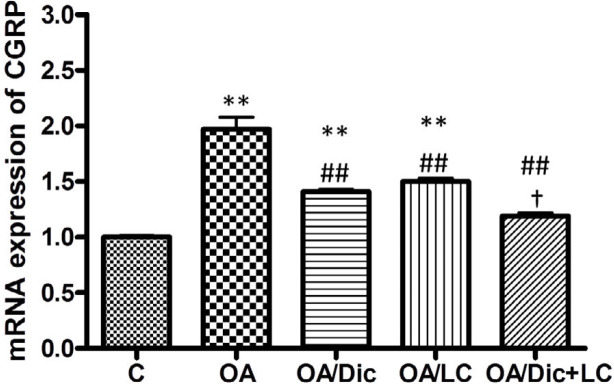
Effect of diclofenac sodium and/or L-carnitine on mRNA expression of calcitonin gene-related peptide. ** *P<*0.001 vs C group; ## *P<*0.001 vs OA group; † *P<*0.05 vs OA/ LC

CGRP expression was significantly higher in the OA group than in the C group (*P*<0.001). The treatment with Dic and/or LC caused a significant decrease in CGRP expression, compared with the OA group (*P*<0.001). The combined treatment with Dic+LC caused a significant decrease in CGRP expression than LC alone (*P*<0.05). There was an insignificant difference between OA/Dic+LC and C groups (*P*>0.05)(Figure 4).

***Histopathological results***

**Figure 5:**
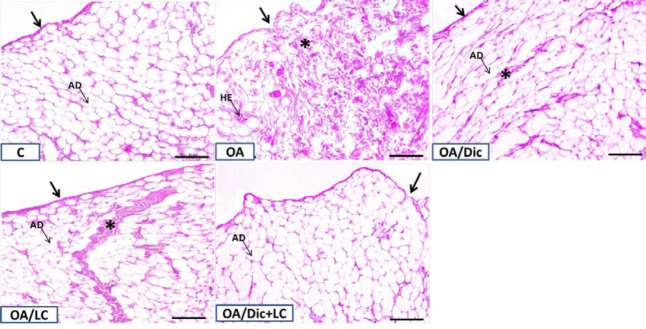
Representative H&E staining of rat synovial membrane of different groups: the membrane of C group consists of thin synovial intima (black arrow), and subintima with predominance of adipocytes (AD). The synovial membrane of OA group appeared moderately thickened with a disorganized intima (black arrow) and, the subintima, excessive fibrous tissue (asterisk) with few adipocytes (AD) and blood vessels full of red blood cells (HE). The synovial membranes of both OA/Dic and OA/LC groups, showed intimal tissue organization (black arrow), although the subintima appeared moderately fibrous (asterisk). OA/Dic+LC group, histopathological changes were considerably ameliorated with mild irregularity of the synovial intimal layer (black arrow) and remarkable reduction in the subintimal fibrous tissue with appearance of abundant adipocytes (AD). Scale bar 50 µM, ×200

**Figure 6:**
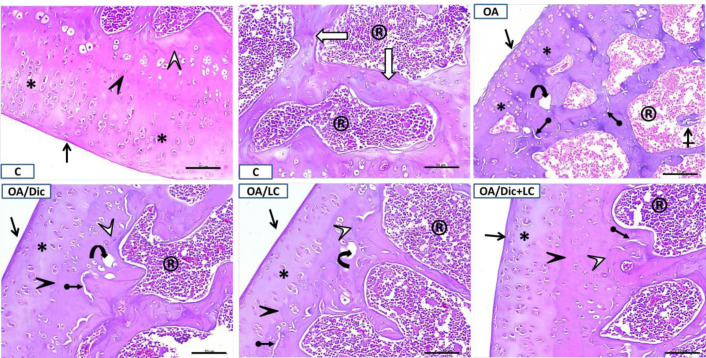
Representative H&E staining of the knee joint of different groups. In the C group, smooth articular surface (black arrow), normal parallel rows of chondrocytes (asterisk) and intact tidemark (black arrowhead) were detected. The subchondral bone showing bone marrow (®) filled with blood-forming elements surrounded by bone trabeculae (white arrow). Note, bony spicules fixing it to cartilage (white arrowhead). In the OA group, irregularity of articular surface (black arrow), hypocellularity (asterisk) with chondrocytes disorganization of articular cartilage and ill-defined tidemark line were detected. Subchondral bone showed cyst formation (curved arrow), fragmentation of the bony trabeculae (crossed arrows) and cracks (arrow with rounded end). Disappeared bony spicules and fibrous tissue formation in the bone marrow space (®) can also be detected. In OA/Dic, OA/LC, and OA/Dic+LC groups, articular cartilage surface was regular (black arrow). Chondrocyte number was increased (asterisk) with intact tidemark (black arrowhead). Few trabecular cracks (arrow with rounded end) were still detected in the subchondral bone. Subchondral cysts (curved arrow) were detected in OA/Dic and OA/LC groups. Scale bar=50 μm×200

The synovial membrane of the knee joint in the control group showed normal characteristics, thin synovial intima, and subintima with a predominance of adipose cells. The articular surfaces were smooth, and the chondrocytes were normally distributed. There were normally distributed bone trabeculae surrounding the bone marrow filled with blood-forming elements with bony spicules that fix it to the cartilage. A single prominent tidemark between non-calcified and calcified parts of the cartilage was detected (Figure 5 and 6).

In the OA group, the synovial membrane appeared moderately thickened with a disorganized intima; in the subintima, the adipose connective tissue was replaced by fibrous tissue. Irregularities and mild fibrillation of the articular surface were observed. There was hypocellularity, chondrocyte disorganization, and ill-defined tidemark. The subchondral bone showed a thinned trabecular wall, cyst formation in addition to fragmentations of the bony trabeculae. Cracks and absence of bony spicules were also observed. The bone marrow elements were replaced with abnormal fibrous tissue (Figures 5 and 6). 

In both OA/Dic and OA/LC groups, the synovial subintima was slightly fibrous; however, the intima was shown to be organized. There was a thick and fairly smooth articular surface in the OA/LC group. The chondrocytes exhibited mild disorganization with scattered shadows of degenerated cells. Faint tidemark line was seen. The subchondral bone showed cyst formation, cracks, and slightly absent bony spicules. On the other hand, histopathological changes were considerably ameliorated in OA/Dic+LC group with mild irregularity of the synovial intimal layer, mild disorganization of chondrocytes, and small cracks in subchondral bone were still detected (Figures 5 and 6).

**Figure 7:**
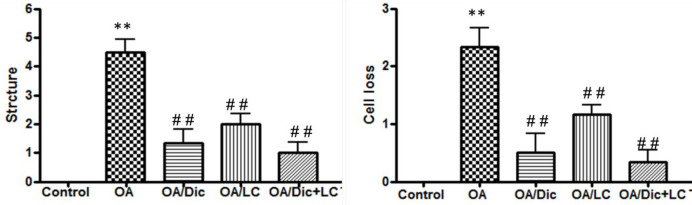
Modified Mankin’s score. ** *P<*0.001 vs C group; ## *P<*0.001 vs OA group

The structural damage and cellular abnormalities were evaluated using the modified Mankin’s score. OA/Dic, OA/LC, and OA/Dic+LC treated groups all showed significantly lower Mankin’s scores than the OA group (*P*<0.001). OA/Dic+LC group showed the lowest scores (Figure 7).

**Figure 8:**
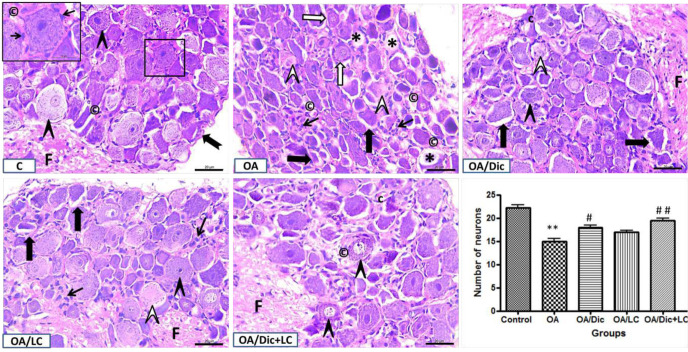
Representative H&E staining of rat dorsal root ganglia of different groups. The dorsal root ganglia of C group showing multiple large and small neurons (arrow heads) with rounded open face nuclei surrounded by a satellite glial cell sheath (thin arrows) and enclosed by a thin connective tissue capsule (notched arrow). Note, few capillaries (©) in the interstitial stroma and nerve fibers close to the ganglion (F) could be detected. The OA group shows shrunken neurons (black arrows) with wide perineural spaces, some neurons show peripheral chromatolysis (white arrows) others show central chromatolysis (white arrow heads). There is empty neural spaces (stars), satellite cell proliferation (thin black arrows) and dilated capillaries (©) in the interstitial stroma. Both OA/Dic and OA/LC groups show multiple cells that appear normal (black arrowhead). A few cells appear distorted and shrunken (thick arrow) and others show central chromatolysis (white arrowhead). In OA/LC group, proliferated satellite cells (thin black arrows) could be noticed in the interstitial stroma. OA/Dic+LC group appears similar to the control group except for some dilated capillaries (©). The number of neurons (F) were dramatically decreased in OA group; ** *P<*0.001 vs C group. This decrease was significantly increased in the OA/Dic and OA/Dic+LC treated groups; # *P<*0.05, ## *P<*0.001, respectively vs OA group. Scale bar 20 µM, ×400

The neurons of DRG of the C group were spherical or ovoid somata. They were aggregated in groups and were variable in size. Large and small neurons were surrounded by a thin connective tissue capsule. The neurons had rounded open face nuclei and a capsule of flat epithelial satellite cells. Few capillaries were observed in the interstitial stroma. Nerve fibers were visible close to the ganglion. DRG of the OA group showed shrunken neurons with wide perineural spaces, some neurons showed peripheral chromatolysis while others showed central chromatolysis. There were empty neural spaces, proliferated satellite cells and dilated capillaries in the interstitial stroma. The number of neurons was dramatically decreased compared with the C group (*P*<0.001). In both OA/Dic and OA/LC groups, shrunken neurons with wide perineural spaces, some neurons with central chromatolysis were still detected. OA/LC group also showed fibrosis in the interstitial stroma. On the other hand, OA/Dic+LC group displayed nearly normal appearance like the C group. The number of neurons was significantly increased in OA/Dic and OA/Dic+LC groups (*P*<0.05 and *P*<0.001, respectively), compared with the OA group (Figure 8).

***Immunohistochemistry***

**Figure 9:**
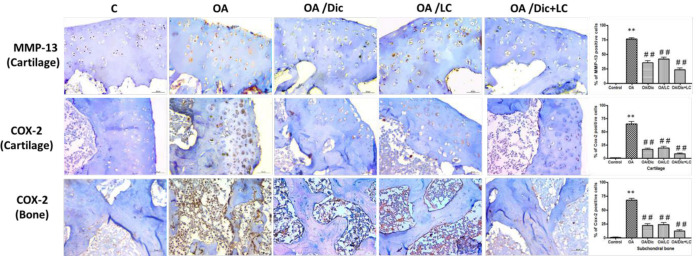
Representative MMP-13 and COX-2 staining of rat knee joint of different groups: MIA injection up-regulated expression of both MMP-13 and COX-2, ** *P<*0.001 vs control group. This increase was significantly decreased in OA/Dic, OA/LC, and OA/Dic+LC groups; ## *P<*0.001 vs OA group. Scale bar 20 µM, ×400

MMP-13 expression in the knee joint was up-regulated by MIA injection (*P*<0.001), and it was down-regulated after treatment with Dic and/or LC (*P*<0.001).The expression of COX-2 was significantly increased in the articular cartilage and the subchondral bone of osteoarthritic rats compared with the control rats (*P*<0.001). Treatment with Dic and/or LC markedly down-regulated expression of COX-2 in both articular cartilage and subchondral bone compared with the OA group (*P*<0.001). The combined treatment showed the largest decrease in both MMP-13 and COX-2 expression (Figure 9).

## Discussion

The management of OA is a great challenge. The current management focuses on the alleviation of osteoarthritic symptoms; however, these symptoms are subsequent to the primary cause, possibly inflammation, and oxidative stress. Thus, in this study, we investigated the efficacy of LC, an antioxidant molecule with an anti-inflammatory effect, as a complementary therapy to Dic treatment in the OA rat model.

The current study showed that intra-articular injection of MIA in the knee joint induced a linear pathology with similarities to human OA features such as chondrocytes damage, synovial inflammation and changes in subchondral bone, which agrees with Bendele (12). The H&E staining of the knee joints showed that coadministration of LC with Dic attenuated the MIA-mediated histopathological alteration in the knee joint. Modified Mankin’s score in the OA/Dic+LC group was significantly lower than in OA/Dic and OA/LC groups. The protective effect of LC in the present study agrees with a previous one (6). 

In accordance with these pathological changes, OA rats showed swollen knee, impaired gait, increased knee bend score, reduced time on rotarod and lower exercise endurance capacity on a treadmill, compared with the C group. The osteoarthritis symptoms remained significant compared with the control rats until the end of the study. This is in agreement with previous studies (6,18). 

Also, the present study showed that coadministration of LC with Dic significantly reduced the severity of knee swelling and improved pain-related behaviors in MIA-induced OA rats, which is in agreement with previous studies (6,24). The combined treatment showed a better effect in improving the knee swelling and the pain-related behaviors and even normalized some values to the normal levels by the end of the experiment.

To explore the underlying mechanism of the antiarthritic and antinociceptive effects of LC, inflammatory, oxidative stress, and bone turnover markers were measured, and immunohistochemistry of MMP-13 and COX-2 in the knee joint were done. Also, histological examination of DRGs and CGRP expression in the spinal cord were assessed to investigate if LC has a central mechanism of action that may be involved in pain relief.

Results have revealed a significant increase of proinflammatory cytokines TNF-α and IL1-β, and the inflammatory mediator PGE2 in MIA-induced OA rats, which is in accordance with the immunohistochemistry results that showed overexpression of COX-2 in the articular cartilage and the subchondral bone. This agrees with another study (15). COX-2 is dramatically up-regulated by inflammation and contributes to PGE2 production, which mediates a number of inflammatory reactions leading to tissue damage (25).

On the other hand, serum levels of TNF-α, IL1-β, and PGE2 were significantly reduced after treatment with Dic and/or LC. These results confirm the anti-inflammatory effect of LC, which has been demonstrated in different experimental models. Tastekin *et al*. reported that TNF-α was significantly reduced by Dic or LC treatment in rats with adjuvant arthritis (26). Also, a study reported a significant decrease of IL1-β by LC treatment in osteoarthritic rats (6). In this study, treatment with Dic and/or LC markedly down-regulated expression of COX-2 in both articular cartilage and subchondral bone, suggesting that LC can reduce pain by reducing the inflammatory effects of increased COX-2. LC had been reported to decrease COX-2 expression in human corneal epithelial cells (27).

Also, osteoarthritic rats showed a significant increase of BALP, a marker of bone turnover, which coincides with a study which reported an increase of BALP in experimentally induced OA in dogs (28). Dic has been reported to decrease BALP in adjuvant-induced arthritic rats, which coincides with our results (29), and LC has been reported to suppress bone turnover in aged ovariectomized rats by decreasing alkaline phosphatase (30).

Oxidative stress plays a role in OA pathophysiology (31). In this study, oxidative stress in osteoarthritic rats, as indicated by significantly higher MDA and significantly lower GSH in the OA group than in the C group, coincides with a previous study (32). The LC administration led to a decrease in MDA levels and increase in GSH levels, confirming the antioxidant effect of LC (5); while, Dic administration led to a significant decrease in MDA level only with an insignificant effect on GSH level, which agreed with Tastekin *et al*. (26). Co-administration of LC with Dic resulted in a better effect in improving oxidative stress than when Dic was administered alone

Oxidative stress is responsible for the activation of MMP, leading to the degradation of the extracellular matrix in cartilage (33). It has been established that chondrocytes in MIA-induced OA rats overexpress MMP-13, which agrees with the immunohistochemistry results in the present study (24). This could be explained by the increase in TNF-α and IL1-β in OA rats (9); whereas, Dic and/or LC partially suppressed the increase in MMP-13 expression. This is in agreement with research that reported that MMP-13 was significantly decreased in cartilage from MIA-induced OA rats treated with acetyl-L-carnitine (24). The combined treatment showed the largest decrease in MMP-13 expression.

The affected peripheral OA region transduces nociceptive signals to central compartments (spinal cord and DRGs) to overproduce inflammatory cytokines and pain-mediators at the level of the sensory neurons and spinal cord (10). In this study, histological examination of DRG in MIA-induced OA rats, showed shrunken neurons with wide perineural spaces and chromatolysis in some neurons, indicating significant neural injury. This is in accordance with others who reported an increase in the expression of ATF-3, a marker for peripheral neuron stress/injury, in DRG cells following intra-articular injection of MIA (34). Dic and LC treatments partially improved these pathological changes in DRG. On the other hand, the combination of Dic+LC reversed the pathological change nearly to the normal, 

CGRP, a nociceptive marker, is known to play a key role in the pathophysiology of OA-induced pain (35). CGRP is released in the spinal cord following the activation of primary afferent neurons and contributes to inflammatory or pain responses (36). Results have revealed that CGRP expression in the L2-5 segment of the spinal cord was significantly higher in OA rats than control rats, and significantly reduced after treatment with Dic and/or LC. Nonsteroidal anti-inflammatory drugs were reported to inhibit stimulated *in vitro* CGRP release from dissected rat trigeminal ganglia (37). To the best of our knowledge, this study is the first to investigate the effect of LC on the expression of CGRP in MIA-induced OA. Results have revealed that LC might alleviate OA-induced pain by inhibiting CGRP expression in the spinal cord. The combined treatment showed a better effect than each treatment alone, and even normalized the values to the normal level.

**Table 1:** Gait score in rats used for pain assessment

Score	Gait description
0	Normal, equal ink staining on both feet
1	Slight limp, toe staining evident and some heel staining for all steps
2	Limping, toes only staining for all steps
3	Dragging and carrying leg, black drag marks from dorsal side of foot present
4	Carrying leg the entire time, no staining from the painful leg or only minor black drag marks

**Table 2:** Modified Mankin’s histological score used for scoring histological injuries of the articular cartilage

**Score**	**Structure (0-6) **
0	Normal
1	Irregular surface, including fissures into the radial layer
2	Pannus
3	Absence of superficial cartilage layers
4	Slight disorganization (cellular row absent, some small superficial clusters)
5	Fissure into the calcified cartilage layer
6	Disorganization (chaotic structure, clusters, and osteoclasts activity).
**Score **	**Cellular abnormality (0-3)**
0	Normal
1	Hypercellularity, including small superficial clusters
2	Clusters
3	Hypocellularity

**Table 3:** Effect of diclofenac sodium and/or L-carnitine on serum levels of oxidative stress and inflammatory markers in mono-iodoacetate induced knee osteoarthritis rat model

**Group** **Parameters**	**C**	**OA **	**OA/Dic**	**OA/LC **	**OA/Dic+LC**
**MDA (nmol/ml)**	3.04±0.25	3.97±0.19^**a^	3.48±0.23^*b^	3.39±0.13^*b^	3.08±0.18^**b*c^
**GSH (mmol/l)**	0.30±0.06	0.07±0.01^**a^	0.14±0.04^*d^	0.26±0.07^**b*c^	0.29±0.05^**b*c^
** IL1-β (pg/ml)**	41.90±3.36	80.40±2.79^**a^	54.80±5.94^**b^	61.20±8.07^**b^	45.40±2.60^**b*c **d^
**TNF-α(ng/ml)**	16.26±1.74	37.74±3.74^**a^	25.50±2.70^**a**b^	29.52±2.40^**a**b^	17.92±1.60^**b**c**d^
**PGE2 (pg/ml)**	386.60±18.18	621.80±40.56^**a^	523.20±22.29^*b^	546.00±51.84^*b^	461.60±14.29^**b*c *d^
**BALP (ng/ml)**	28.74±3.22	39.50±1.96^**a^	34.28±1.23^*b*d^	29.00±2.12^**b*c^	29.34±1.71^**b*c^

Values are presented as mean±SD (n=10). * *P<*0.05, ** *P<*0.001

a: significant vs C, b: significant vs OA, c: significant vs OA/Dic, d: significant vs OA/LC

MDA: malondialdehyde, GSH: reduced glutathione, IL-1β: interleukin-1 beta, TNF-α: tumor necrosis factor-alpha, PGE2: prostaglandin-E2, BALP: bone specific alkaline phosphatase, C: Control, OA: Osteoarthritis, OA/Dic: Osteoarthritis/Diclofenac sodium treated, OA/LC: Osteoarthritis/L-carnitine treated, OA/Dic+LC: Osteoarthritis/Diclofenac sodium+L-carnitine treated

C: Control, OA: Osteoarthritis, OA/Dic: Osteoarthritis/Diclofenac sodium treated, OA/LC: Osteoarthritis/L-carnitine treated, OA/Dic+LC: Osteoarthritis/Diclofenac sodium+L-carnitine treated

* *P<*0.05, ** *P<*0.001 vs C group; # *P<*0.05, ## *P<*0.001 vs OA group

C: Control, OA: Osteoarthritis, OA/Dic: Osteoarthritis/Diclofenac sodium treated, OA/LC: Osteoarthritis/L-carnitine treated, OA/Dic+LC: Osteoarthritis/Diclofenac sodium + L-carnitine treated

C: Control, OA: Osteoarthritis, OA/Dic: Osteoarthritis/Diclofenac sodium treated, OA/LC: Osteoarthritis/L-carnitine treated, OA/Dic+LC: Osteoarthritis/Diclofenac sodium + L-carnitine treated

C: Control, OA: Osteoarthritis, OA/Dic: Osteoarthritis/Diclofenac sodium treated, OA/LC: Osteoarthritis/L-carnitine treated, OA/Dic+LC: Osteoarthritis/Diclofenac sodium + L-carnitine treated

C: Control, OA: Osteoarthritis, OA/Dic: Osteoarthritis/Diclofenac sodium treated, OA/LC: Osteoarthritis/L-carnitine treated, OA/Dic+LC: Osteoarthritis/Diclofenac sodium + L-carnitine treated

C: Control, OA: Osteoarthritis, OA/Dic: Osteoarthritis/Diclofenac sodium treated, OA/LC: Osteoarthritis/L-carnitine treated, OA/Dic+LC: Osteoarthritis/Diclofenac sodium + L-carnitine treated

C: Control, OA: Osteoarthritis, OA/Dic: Osteoarthritis/Diclofenac sodium treated, OA/LC: Osteoarthritis/L-carnitine treated, OA/Dic+LC: Osteoarthritis/Diclofenac sodium + L-carnitine treated

C: Control, OA: Osteoarthritis, OA/Dic: Osteoarthritis/Diclofenac sodium treated, OA/LC: Osteoarthritis/L-carnitine treated, OA/Dic+LC: Osteoarthritis/Diclofenac sodium + L-carnitine treated

C: Control, OA: Osteoarthritis, OA/Dic: Osteoarthritis/Diclofenac sodium treated, OA/LC: Osteoarthritis/L-carnitine treated, OA/Dic+LC: Osteoarthritis/Diclofenac sodium + L-carnitine treated

## Conclusion

 L-Carnitine has a protective effect via anti-inflammatory and antioxidant mechanisms. Also, LC can alleviate pain at central and peripheral levels. LC plus Dic is a more effective therapy than Dic alone in improving OA pathogenesis and symptoms. Thus, LC is a promising complementary therapy to Dic in OA management.
